# Curcumin, a Natural Antioxidant, Acts as a Noncompetitive Inhibitor of Human RNase L in Presence of Its Cofactor 2-5A *In Vitro*

**DOI:** 10.1155/2014/817024

**Published:** 2014-09-02

**Authors:** Ankush Gupta, Pramod C. Rath

**Affiliations:** Molecular Biology Laboratory, School of Life Sciences, Jawaharlal Nehru University, New Delhi 110067, India

## Abstract

Ribonuclease L (RNase L) is an antiviral endoribonuclease of the innate immune system, which is induced and activated by viral infections, interferons, and double stranded RNA (dsRNA) in mammalian cells. Although, RNase L is generally protective against viral infections, abnormal RNase L expression and activity have been associated with a number of diseases. Here, we show that curcumin, a natural plant-derived anti-inflammatory active principle, inhibits RNase L activity; hence, it may be exploited for therapeutic interventions in case of pathological situations associated with excess activation of RNase L.

## 1. Introduction

The 2′,5′-oligoadenylate- (2-5A-) dependent ribonuclease L (RNase L) is one of the key antiviral enzymes of the interferon-inducible 2-5A pathway of mammalian cells. Upon virus infection or interferon (IFN) treatment, a group of enzymes collectively termed as 2′,5′ oligoadenylate synthetases (2-5OAS), activated by double-stranded RNA (dsRNA), often produced as viral replication intermediates, synthesize unique and labile cofactor called 2′,5′-oligoadenylates (2-5A) from ATP. This 2-5A cofactor binds to latent, monomeric RNase L and converts it into an active, dimeric enzyme, which degrades both viral and cellular single-stranded RNAs, thus eliminating both virus and virus-infected cells. RNase L is a 741 a.a. protein (~83 kDa) and has an interesting arrangement of the structural and functional domains. The N-terminal region consists of 9 ankyrin repeats (l-9, 9th one incomplete) often involved in protein-protein/protein-nucleic acid interactions, while the C-terminal region consists of a protein-kinase- (PK-) homology domain, a cysteine-rich region and a ribonuclease domain [1]. Functions of the PK-homology and cysteine-rich domains are uncertain. RNase L undergoes conformational switching as it gets activated by binding of 2-5A to the ankyrin repeats 2–4 [2].

Interestingly, RNase L gene (*RNASEL*) has been identified as a human prostate cancer susceptible (HPC1) locus and has been suggested to function as a tumor suppressor by causing apoptosis of mammalian cells through RNA degradation. Point mutations in RNase L (e.g., R462Q) can potentially decrease its RNase activity and possibly increase susceptibility to prostate cancer [3]. Recent reports suggest that not only RNase L is a marker for HPC but also germline mutations in* RNASEL* may predict an increased risk of head neck, uterine, cervix, and breast carcinoma [4]. In addition, deregulation in 2-5A pathway has been documented in immune cells of chronic fatigue syndrome (CFS) patients characterized by abnormally upregulated OAS and RNase L activities and by the presence of a low molecular weight (LMW) 2-5A-binding protein of 37 kDa related to RNase L produced by proteolytic degradation of the wild type protein. This protein escapes the normal regulation of RNase L leading to a cascade of unwanted cellular events, probably due to preferential production of 2-5A dimers instead of higher oligomers. The origin of the 2-5OAS dysregulation and production of these 2-5A dimers still remain speculative and the 37 kDa RNase L protein might be a result of improper activation and cleavage due to these dimers [5]. Moreover, induction of RNase L protein by stress-inducing agents such as double stranded RNA (poly rI: rC), chemotherapeutic anticancer drugs, H_2_O_2_, calcium chloride, and TNF-α in mammalian cells also indicates a possible role for RNase L in stress-responsive cellular functions [6]. Similarly, elevated expression of RNase L protein and mRNA in colorectal adenocarcinomas suggests its involvement in early events of colorectal carcinogenesis [7]. Identification of upregulated genes in the scrapie-infected brain tissue by subtractive hybridization revealed the upregulation of 2′,5′ oligoadenylate synthetase as one of the many interferon-inducible genes and it suggested apoptotic loss of neuronal cells probably by hyperactivation of RNase L [8]. Thus, considering the broad range of protective cellular functions of RNase L starting from the antiviral, antiproliferative, apoptotic, antineoplastic, and immunomodulatory effects to its role in cellular RNA metabolism, translational regulation [9], stress response, and antibacterial immunity [10], we decided to study the effect of the naturally occurring, nontoxic, and polyphenolic antioxidant curcumin on RNase L activity.

Curcumin, an orange-yellow pigment obtained from the dried rhizomes of the perennial herb* Curcuma longa *Linn. (family Zingiberaceae) is commonly used as a spice in Indian food. Curcumin, chemically identified as 1,7-bis-[4 hydroxy-3-methoxy]-1,6 heptadiene-3,5-dione or diferuloylmethane, has been traditionally used primarily as an curative agent against infections by pathogens and inflammation due to injury; recently many of its therapeutic effects have been confirmed as antioxidant, anti-inflammatory, anticarcinogenic, antimicrobial, hepatoprotective, thrombosuppressive, cardiovascular protective agent (protection against myocardial infarction), hypoglycemic, antiarthritic (protection against rheumatoid arthritis), and neuroprotective [11]. In this paper, we report that purified curcumin acts as a noncompetitive inhibitor of the enzymatic activity of full-length (741 a.a.) recombinant human RNase L in the presence of its cofactor, 2-5A* in vitro*. This inhibitory effect of curcumin on full-length RNase L activity may be extrapolated as a therapeutic approach for the treatment of chronic fatigue syndrome patients, in the early development of colorectal tumors and loss of neuronal cells in scrapie-infected brain tissues as well as many immune dysfunctions due to excessive RNase L activity.

## 2. Materials and Methods

### 2.1. Reagents, Plasmids, Oligonucleotides, and* E. coli* Strains

*E. coli* strains DH5α and XL-1 blue were used as host cells for cloning and expression of recombinant human RNase L, respectively. The LB-medium and LB-agar plates were supplemented with 100 *μ*g/mL of ampicillin. The pBluescript II SK- (+) vector (Stratagene, U.S.A.) was used for DNA cloning and pGEX 2TK-vector (Amersham, U.S.A.) was used for protein expression. Oligonucleotides were commercially synthesized (Microsynth, Switzerland) and Pfu-DNA polymerase (Finnzymes) was used for PCR amplification of the RNase L cDNA. Glutathione-agarose beads, IPTG, and reagents used for RNase-activity assay were from Sigma Aldrich, USA. The human RNase L cDNA (pZC5) and the 2-5A cofactor [pppA(2′p5′A)_3_ (tetramer)] [12] used in the ribonuclease assay were kind gifts from Professor R. H. Silverman, Cleveland Clinic Foundation, OH.

### 2.2. pGEX-hRNase L Construct Preparation, Expression, and Purification of GST-hRNase L

The cloning, expression and purification of the GST-RNase L fusion protein was performed as described in detail in our earlier work [13]. Briefly, the blunt-ended PCR fragment amplified from pZC5 [12] plasmid encoding the full-length human RNase L was kinased and ligated into pBluescript II SK- (+) vector at Sma I site generating pBS-hRNL plasmid. The Bam HI fragment containing RNase L from pBS-hRNL was subcloned into Bam HI site of pGEX-2TK-vector generating pGEX-hRNL clones to express the RNase L as a GST-RNase L fusion protein. The recombinant plasmids were transformed into* E. coli* DH5α cells to prepare plasmid DNA and* E. coli* XL-1 blue cells to express the GST-RNase L fusion protein.

Briefly, for GST-RNase L expression, a freshly transformed colony of pGEX-hRNL/XL-1 blue cells grown overnight at 37°C, was inoculated in primary culture of 10 mL LB^Amp^ and grown overnight at 37°C with shaking at 220 rpm. A secondary culture of 100 mL LB^Amp^ 2% glucose was inoculated with 3% inoculum from primary culture and incubated at 37°C/220 rpm until the O.D_600 _nm of the culture reached ~0.6–0.8. The cells were then induced at 18°C/220 rpm for 18–21 hours with 0.3 mM IPTG after prior reduction of the culture temperature to 18°C. The cells were pelleted at 6000 rpm for 10 min at 4°C and the cell pellet from 200 mL culture was washed once in 10 mL buffer A [phosphate buffered saline (PBS), 10% glycerol, 1 mM EDTA, 0.1 mM ATP, 5 mM Mgcl_2_, 14 mM 2-mercaptoethanol, 2 *μ*g/mL leupeptin, and 2 mM PMSF]. The washed cell pellet was resuspended in 10 mL buffer A supplemented with lysozyme (100 *μ*g/mL) and lysed by sonication on ice at 18 micron for 15 sec. five times. The protein was solubilized by incubation on a rocking platform at 4°C for 30 min after addition of 1% (v/v) Triton X-100 and the supernatant was collected after centrifugation at 13000 rpm for 15 min. at 4°C. Purification of the fusion protein was performed in batch affinity as per the manufacturer's instructions of glutathione agarose (Sigma Aldrich, USA), with minor modifications. The glutathione agarose [250 *μ*L of a 50% (v/v) slurry preequilibrated with buffer A] was incubated with clarified cell lysate on ice on a rocking platform at 4°C for 1 hour. After washing the protein-bead mixture three times with 10 mL of buffer A and centrifugation at 3000 rpm to recover the bound fraction, the fusion proteins were eluted in 0.5 mL of buffer B [20 mM reduced glutathione, 50 mM Tris. Cl (pH 8.8), 100 mM KCl, 0.1% Triton X-100, 2 mM EDTA, 14 mM 2-mercaptoethanol, 0.2 mM ATP, 10 mM Mgcl_2_, and 1 *μ*g/mL leupeptin] three times with incubation at 4°C for 15 min. each time. 20% sterile glycerol was added to the eluted fraction and small aliquots were stored at −80°C. For total intrinsic fluorescence studies, the buffer exchange and concentration of the affinity-purified protein was performed on Centricon-10 column (Millipore) in buffer C (50 mM Tris. Cl pH 7.5, 200 mM NaCl, and 5 mM 2-mercaptoethanol). The quality of the purified protein was monitored by SDS-PAGE followed by Coomassie blue R-250 staining [13]. The protein concentration was determined by Bradford assay using BSA as standard.

### 2.3. RNase-Activity Assay of GST-hRNase L

The RNase L activity assay was performed as per our earlier work [13], which is broadly based upon the RNase L assay performed by Yoshimura et al. [14]. Mouse kidney total RNA was prepared by using the LiCl precipitation method [15]. The RNA-degradation reaction mixture was set up in a volume excluding the RNA substrate in the reaction buffer D [22.2 mM Tris. Cl (pH 7.5), 11.1 mM magnesium acetate, 8.9 mM 2-mercaptoethanol, and 0.11 M KCl], ATP (0.11 mM), GST-hRNase L (50 ng/20 *μ*L), 2-5A cofactor (10 nM), curcumin (variable concentration/3 *μ*L), and double distilled, deionized, sterile water. A 20 mM main stock of curcumin was prepared in absolute ethanol and then substocks from 0.034 mM to 1 mM were prepared in sterile deionized water from the main stock. To ensure negligible ethanol concentrations, 3 *μ*L curcumin/20 *μ*L reaction was added from different substocks, which accounted for a final concentration of ethanol from 82.5 × 10^−6^% to 7.5 × 10^−3^% (v/v). This reaction mixture was preincubated on ice for 30–45 min. for dimerization and activation of GST-hRNase L. Finally, variable amount of mouse kidney total RNA (1 *μ*g/*μ*L) was added to the reaction mixture to make the final volume to 20 *μ*L and incubated at 30°C for 10 min. The reaction was terminated by adding 4.0 *μ*L of 6 X RNA loading dye [10 mM Tris.cl (pH7.5), 0.1% bromophenol blue, 60 mM EDTA, and 60% glycerol] and 12 *μ*L (50% reaction) of each sample was resolved by 1.2% agarose-TBE gel electrophoresis stained with 0.5 *μ*g/mL ethidium bromide. Quantitation of RNA degradation (RNase activity of purified GST-hRNase L) was carried out by measuring the intensity of the residual intact bands of 28S and 18S rRNAs in each reaction and subtracting it from that of the control reaction, which did not contain 2-5A cofactor (% 28S and 18S rRNA degraded). The quantity and quality of RNA was measured by taking the absorbance at 260 nm and the corresponding ratios A_260/280_ and A_260/230_, respectively. The percentage RNA degradation was converted into pmol/min of the RNA substrate degraded in 20 *μ*L reaction volume as per our earlier work [13]. Densitometric measurements were carried out by Alpha imager 3400 and AlphaEase FC software.* Ki* of curcumin binding to RNase L was determined by performing a nonlinear regression analysis by fitting the acquired data to noncompetitive equation for inhibitor binding using GraphPad Prism (version 5.04 for Windows, GraphPad Software, San Diego, CA).

### 2.4. Total Intrinsic Fluorescence Measurement for Curcumin Binding with RNase L

The homogeneously purified human RNase L protein obtained after affinity purification and concentration by Centricon-10 was used for studies on intrinsic fluorescence with the help of Varian fluorescence spectrophotometer (Cary Eclipse) at room temperature. Fluorescence excitation was set at 280 nm, and emission spectra were recorded from 300 to 500 nm at a bandwidth of 1 nm. The excitation and emission slit widths were set at 5 nm, respectively. Each recorded spectrum represents an average of three scans. The spectra were recorded at protein concentration of 1 *μ*M in the presence of 50 mM Tris. Cl (pH 7.5), 200 mM NaCl, and 5 mM *β*-mercaptoethanol with increasing concentration of curcumin (0.25–16 *μ*M). [Curcumin]/[RNase L] molar ratio was varied between 0.125 and 32. From a 20 mM main stock of curcumin prepared in absolute ethanol, substocks of 2 mM and 0.2 mM were prepared in sterile deionized water and added into the final reaction mix of 300 *μ*L such that ethanol concentration never exceeded 0.4 × 10^−3^% (v/v). Control experiments with RNase L and similar ethanol concentrations as the main reaction proved the effect of the organic solvent to be undetectable on the intrinsic fluorescence of the protein. The background emission was eliminated by subtracting the signal of the buffer alone from the test samples. The change in intrinsic fluorescence intensity [(*F*_0_ − *F*)/*F*_0_] at 340 nm with increasing curcumin concentration and in absence of curcumin was plotted, and the* Kd *values were determined from a nonlinear least square regression analysis of the titration data by fitting of saturation curve to the Hill slope using GraphPad Prism (version 5.04 for Windows, GraphPad Software, San Diego, CA).

### 2.5. Docking of Curcumin to the Human RNase L Crystal Structure

Molecular docking experiments were conducted to map the possible curcumin binding sites to human RNase L. For docking calculations recently published crystal structure of Human RNAse L (PDB Id: 4OAU) [16] and 3D structure model of curcumin (CID: 969569) were used. Potential binding sites in human RNAse L were identified using metaPocket 2.0 server (http://projects.biotec.tu-dresden.de/metapocket/) [17]. Molecular docking experiment was performed using AutoDock Vina [18], which automatically calculates the grid maps. Each time when the docking was performed, the grids were centred so as to cover the pockets. Since curcumin can undergo keto-enol tautomerism in solution, the enolic form can exist in different* cis* and* trans* isomeric forms depending on temperature, polarity, or hydrogen bonding nature of solvents [19]; hence, 15 different conformers for curcumin were generated using Discovery Studio 3.5 (Accelrys Inc., San Diego, CA, USA). For each conformer 10 cycles of molecular docking was performed. For every torsion (for flexible bonds) of curcumin, all over 150 cycles of docking calculations were done by Vina at every cluster of pocket (binding site 1–5). Chimera 1.8, Pymol, and AutoDock visualizer were used for analysis of structural and docking results.

## 3. Results

**Figure 1:**
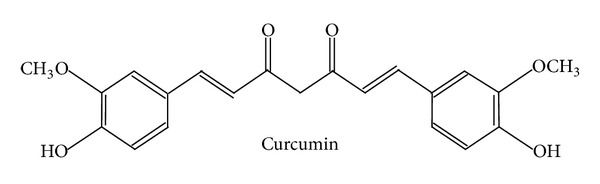
Chemical structure of curcumin.

**Figure 2:**
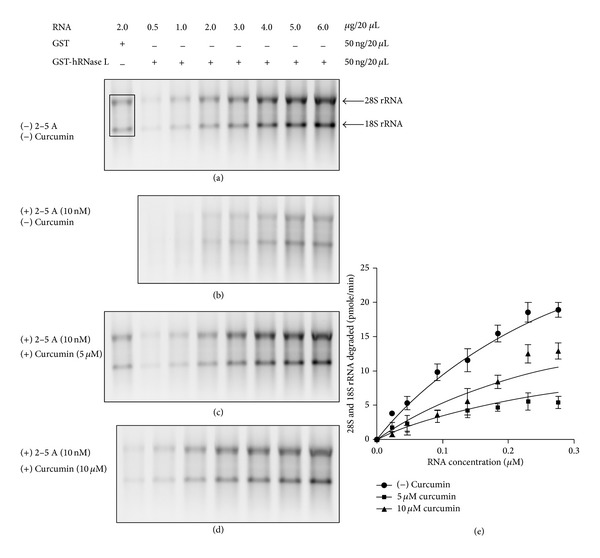
Effect of curcumin on RNase L activity. 1.2% agarose-TBE gel showing inhibition of RNase L activity with purified GST-hRNase L, 2-5A (10 nM), and in absence and presence of 5 *μ*M and 10 *μ*M curcumin, respectively. Panel (a): control lanes with no 2-5A cofactor, panel (b): activity in presence of 2-5A cofactor and no curcumin, panels (c) and (d): inhibition in activity in presence of 5 *μ*M and 10 *μ*M curcumin, respectively. Panels (a) and (b) as well as panel (c) and (d) are parts of the same gels 1 and 2 (both 18 lanes) with control lanes of 2 *μ*g RNA loaded onto each gel for intensity normalization and empty lanes for background subtraction. RNase L activity was calculated from the decrease in the intensity of ribosomal RNA bands, which were converted into pmol/min RNA degraded as mentioned in materials and methods. (e) Plot generated by fitting the data to nonlinear regression analysis, which gave a *Ki* of 4.136 *μ*M. Square bracket encompassing 28S and 18S rRNA bands depict the area used for intensity calculations.

**Figure 3:**
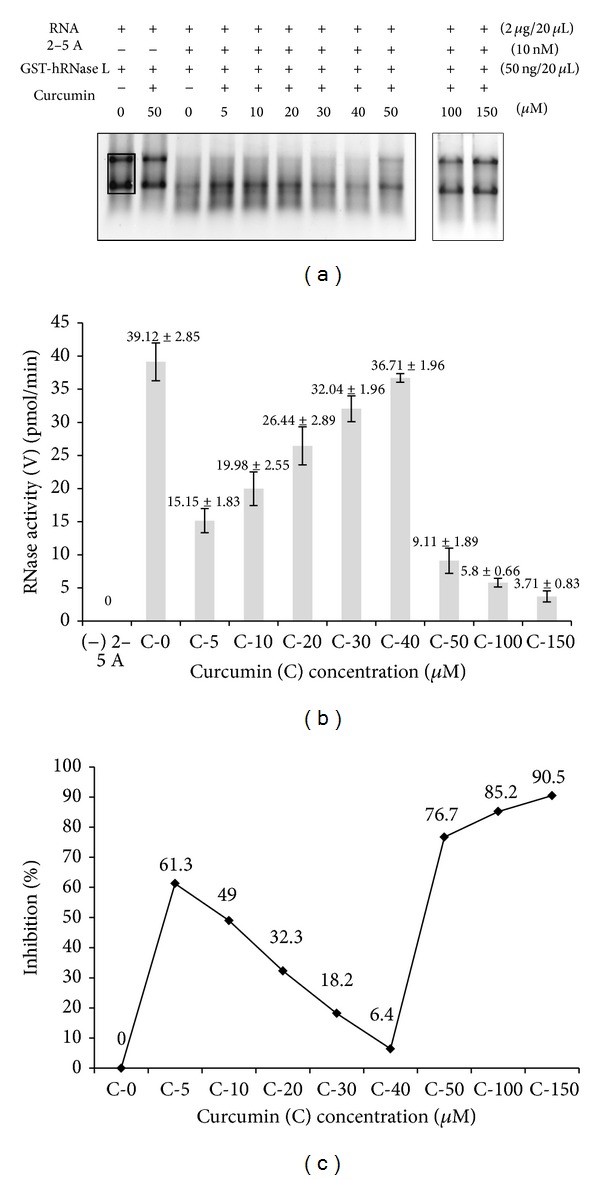
RNA-degradation activity assay showing the effect of increasing concentration of curcumin on RNase L activity. (a) 1.2% agarose-TBE gel showing RNA degradation profile at 30°C for 10 min. RNA 92 nM (2 *μ*g/20 *μ*L) as mouse kidney total RNA; 2-5A (10 nM); GST-hRNase L (22.5 nM each). (b) Graph depicting quantitative degradation of 28S and 18S rRNAs for GST-hRNase L with increasing concentrations of curcumin (5 *μ*M to 150 *μ*M) expressed as pmol/min RNA degraded. (c) Graph depicting percentage inhibition in GST-hRNase L activity with increasing concentrations of curcumin.

RNase L is an IFN-inducible endoribonuclease of the innate immune system of vertebrates. Curcumin has been reported to possess a number of therapeutic properties against various diseases [20]. In this report, we examined the effect of curcumin (1,7-bis-[4 hydroxy-3-methoxy]-1,6 heptadiene-3,5-dione) (Sigma C-1386) (Figure 1) on the recombinant human RNase L activity against total cellular RNA from mouse kidney by scoring 28S and 18S rRNA degradation. The recombinant GST-hRNase L protein produced had the theoretical* pI* and molecular weight of 6.27 and 110.63 kDa, respectively. The effect of various concentrations of curcumin was checked on 22.5 nM (50 ng) of purified GST-hRNase L in presence of 10 nM 2-5A cofactor, which was required to dimerize RNase L. Interestingly, RNase L activity was preincubated with 2-5A at different concentrations of curcumin for 45 min. followed by incubation with increasing concentrations of mouse kidney total RNA for 10 min. at 30°C. Figures 2(a) and 2(b), which depict the negative control reactions without the cofactor and with cofactor, respectively, as well as Figures 2(c) and 2(d), which depict the activity for 5 *μ*M and 10 *μ*M curcumin, respectively, are parts of the same gels 1 and 2 (both 18 lanes) with control lanes of 2 *μ*g RNA loaded onto each gel for intensity normalization and empty lanes for background subtraction were used for data acquisition. Figures 2(a) to 2(d) depict the inhibitory effect of lower concentrations of curcumin (5 *μ*M and 10 *μ*M) on GST-hRNase L activity and the inhibition occurred in a dose-dependent manner. The inhibition kinetics was then analyzed by performing nonlinear regression analysis by fitting the acquired data to noncompetitive equation for inhibitor binding (Figure 2(e)), which indicated that curcumin acts as a noncompetitive inhibitor of RNase L. After the nonlinear regression analysis with lower concentrations of curcumin, a* Ki* value of 4.136 *μ*M was obtained (Figure 2(e)). Then the effect of a broad range of curcumin concentrations was examined on fixed concentrations of GST-hRNase L (22.5 nM), 2-5A (10 nM), and RNA (~92 nM or 2 *μ*g/20 *μ*L of reaction mix) (Figures 3(a) and 3(b)). The inhibitions at 5, 10, 20, 30, and 40 *μ*M curcumin were observed as 61.3%, 49%, 32.3%, 18.2%, and 6.4%, respectively. Interestingly, it showed maximum inhibition with lower concentrations of curcumin, that is, 5 *μ*M and 10 *μ*M, but as the concentration of curcumin was increased, the inhibition was relieved till 40 *μ*M. Further, as the concentration of curcumin was raised to 50 *μ*M, 100 *μ*M and 150 *μ*M, there was again an increase in the inhibition to 77%, 85%, and 90.5%, respectively, (Figure 3(c)) showing biphasic inhibition kinetics by curcumin. This indicated that there is probably a conformational change of RNase L in the presence of 5 to 40 *μ*M curcumin, which relieves the inhibition of the enzyme and there is also again a conformational switching occurring between 40 and 50 *μ*M and above curcumin concentrations that again shows >90% inhibition at the given concentration of the enzyme, cofactor (2-5A), and the substrate RNA.

### 3.1. Binding of Curcumin to Human RNase L

**Figure 4:**
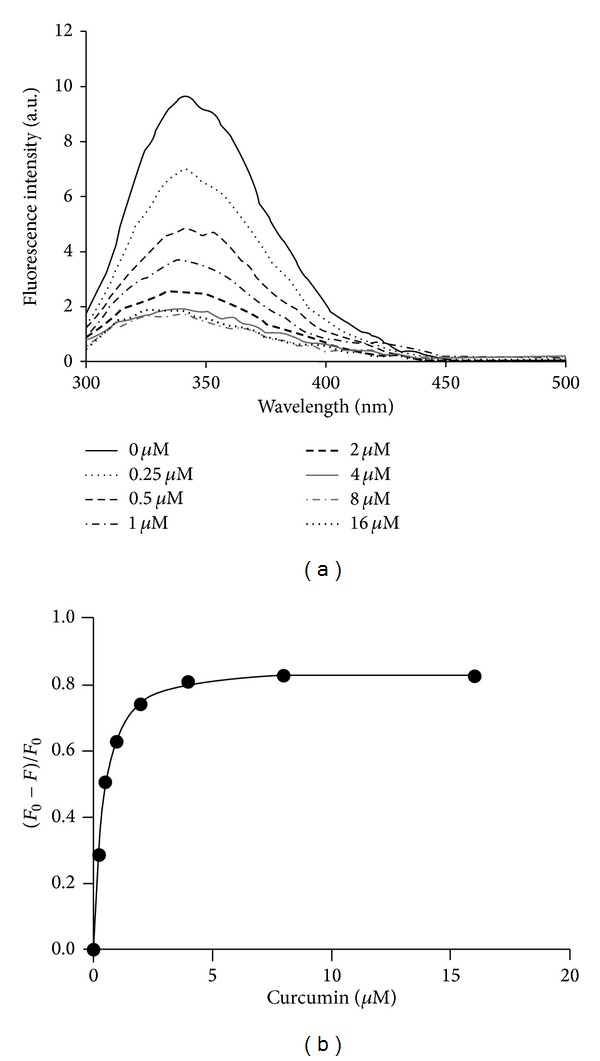
Curcumin binds to human RNase L directly with high affinity. (a) Effect of curcumin on the total intrinsic fluorescence of the purified human RNase L (GST-RNase L) protein (1 *μ*M) in buffer (50 mM Tris.Cl (pH 7.5), 200 mM NaCl, and 5 mM *β*-ME). Fluorescence emission spectra indicate quenching and blue shift of the fluorescence intensity of human RNase L during the titration by increasing curcumin concentration (0.25 *μ*M to 16 *μ*M). (b) Saturation curves were generated from the intrinsic fluorescence data by plotting the change in fluorescence intensity at 340 nm as a function of increasing curcumin concentration, where *F*_0_ and *F* are fluorescence intensity in the absence and presence of curcumin, respectively. *K*_*D*_, determined by the fit of fluorescence intensity on the Hill plot, was 0.397 ± 0.018 *μ*M. Excitation wavelength of 280 nm was used.

Binding of curcumin to several proteins have been studied earlier using biophysical techniques like intrinsic fluorescence quenching and circular dichroism studies [19, 22, 23]. To investigate the binding affinity of curcumin with the human RNase L protein, we used total intrinsic fluorescence as well as fluorescence quenching technique and measured the *K*_*D*_ for the binding (Figures 4(a) and 4(b)). Curcumin quenched the total intrinsic fluorescence of RNase L in a concentration-dependent manner. The intrinsic fluorescence of the human RNase L protein is due to the presence of seven tryptophan, eighteen tyrosine and twenty-eight phenylalanine residues.  Figure 4(a) depicts the plot of fluorescence quenching data for human RNase L in the presence of increasing concentrations of curcumin (0.25 *μ*M to 16 *μ*M). The emission maximum of human RNase L in absence of curcumin was observed at 340 nm (9.87 a.u.), which was blue shifted from 340 nm to 332 nm in presence of 2–4 *μ*M curcumin (2.72 and 2.02 a.u., resp.). The emission maximum further blue shifted to 331 nm from 340 nm at 8 *μ*M curcumin (1.8 a.u.) but at 16 *μ*M curcumin, the emission maximum came close to that of the native protein at 337 nm (1.99 a.u.) (Figure 4(a)). The highest blue shift of 9 nm was observed until the concentration of curcumin was increased to 8 *μ*M and then it reverted close to its native emission maximum with a 3 nm blue shift at 16 *μ*M curcumin. This data clearly indicates that upto 8 *μ*M curcumin, there is strong binding and internalization of curcumin in the hydrophobic region of RNase L, but above 8 *μ*M, there is a change in conformation probably due to binding of curcumin to different residues at some other sites. The *K*_*D*_ value of 0.397 ± 0.018 *μ*M was determined by fitting the change in fluorescence intensity data at 340 nm by using Hill equation (Figure 4(b)).

### 3.2. Docking of Curcumin to the Human RNase L

**Figure 5:**
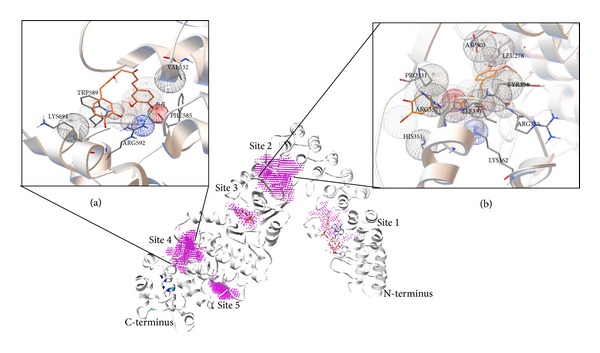
Structure of human RNAse L (4OAU)-curcumin complex obtained after docking analysis as described in materials and methods. Sites filled with magenta dots are pockets predicted as potential binding sites for curcumin. Site 1 is occupied by 2-5A and site 3 by ADP in native structure. Residues highlighted in blue, cyan, and green are active site residues showing position of active site [21]. Inset Figures 5(a) and 5(b) show interaction of curcumin (backbone C in orange color) at site 4 and site 2, respectively. Oxygen atoms are shown in red and nitrogen in blue. Green dotted lines indicate possible H-bonds. Wireframe spheres are interaction spheres between atoms of ligand and receptor.

In order to determine the possible curcumin binding sites in human RNase L, we conducted the molecular docking experiment with the recently solved crystal structure of human RNase L. RNase L forms the intertwined crossed homodimeric structure stabilized by ankyrin repeat (ANK) and kinase homology (KH) domains thereby positioning the kinase extension nuclease (KEN) domains in each subunit for RNA substrate recognition [16]. The pseudokinase domain is involved in 2-5A sensing, dimerization, nucleotide binding, and ribonuclease functions, which explains the evolutionary adaptability of this eukaryotic protein-kinase fold [21]. Thirteen potential binding sites in human RNAse L were identified using metaPocket 2.0 server (http://projects.biotec.tu-dresden.de/metapocket/) [17] for the RNase L protein (3OAU) and these 13 sites formed 5 clusters of pocket (Site 1–5) (Figure 5). Docking calculations explored two energetically equivalent sites (binding site 2 and 4) where docking energy at site 4 was 0.9 kcal/mole higher (−8.5 kcal/mol) than the lowest energy result at site 2 (−7.6 kcal/mol) (Table 1). The computational analysis revealed that according to binding energy, the most favorable docking solutions suggested placement of curcumin into the hydrophobic-binding pocket (site 4) of RNase L (Figure 5). Several docking solutions with a comparable score are also mentioned (Table 1). The site 4 is situated at interlobe pocket of kinase homology domain and RNase domain near to the active site where Val-532, Phe-585, Trp-589, Arg-592, and Lys-694 residues come close enough for making interactions. Arg-592 is involved in H bonding. The site 2 is situated in the pocket formed by C-terminus of ankyrin repeat domain and extended loop and helix region of N-terminus lobe of kinase homology domain. The directionality is much less pronounced at site 2 with no hydrogen-bonding interactions. Amino acids involved in making interaction at site 2 are Leu-278, Asp-303, Pro-331, His-351, Arg-352, Ile-353, Arg-355, and Lys-362.

## 4. Discussion

We report that curcumin is a specific, potent, and noncompetitive inhibitor of recombinant human RNase L at fairly low concentrations, that is, 5 *μ*M and 10 *μ*M. An active RNase L can cause extensive degradation of cellular RNAs leading to apoptosis; hence, the enzyme has to be critically regulated at the level of either its synthesis or activity. The expression of RNase L under normal conditions is very limited in most of the cells and tissues; however, very little is known about the regulation of the RNase L activity. RNase L undergoes subtle conformational changes with respect to its N-terminal 2-5A-binding, dimerization, and hence its activation [2]. N-terminal deletion of 1–335 a.a. leads to a constitutively active RNase L protein [24], while deletion of C-terminal ribonuclease domain makes it a dominant negative molecule inhibiting the activity of wild type RNase L [25]. Point mutants of RNase L like K392R and R462Q in the protein-kinase homology domain and Y712A and F716A in the ribonuclease domain render the molecule partially or completely inactive, again emphasizing its inherent conformational switching nature [26].

In order to combat the mechanism by which viruses evade the IFN pathways, small molecule activators of RNase L with antiviral nature were discovered, which had similar mechanistic action towards RNase L activation as 2-5A and they activated RNase L* in vitro* at micromolar concentrations [27]. Curcumin is a potent inhibitor of TNF-mediated, phorbol ester, and hydrogen-peroxide-mediated activation of NF-*κ*B and it inhibits phosphorylation and degradation of I*κ*Bα hence the translocation of p65 subunit to the nucleus [28]. Curcumin directly interacts and inhibits various proteins, for example, cyclooxygenase-2 (COX-2), lipoxygenase (LOX), glycogen synthase kinase (GSK)-3*β*, phosphorylase-3 kinase, xanthine oxidase, N-aminopeptidase, amyloid protein, human a1-acid glycoprotein, autophosphorylation activated protein kinase, DNA polymerase, focal adhesion kinase (FAK), pp60 src tyrosine kinase, thioredoxin reductase (TrxR), tubulin, topoisomerase II, ubiquitin isopeptidase, and toll-like receptor- (TLR-) 4 [11]. Previous studies have demonstrated that curcumin accelerates oxidative folding of cysteine-rich proteins like RNase A through multiple mechanisms probably by stabilizing the disulphide bonds, which do not involve redox chemistry [29]. Recently, we have reported a convenient method for expression, purification, and characterization of the recombinant human RNase L as a GST-RNase L fusion protein [13]. This will help in identifying small molecular weight reagents binding to RNase L and modulating its activity. We have also identified several RNase L-interacting proteins from natural mammalian tissue like mouse spleen [30]. These RNase L-interacting proteins may reveal natural biological role of RNase L in mammalian cells and tissues.

Curcumin basically interacts with the proteins via offering moieties for hydrogen bonding and also the two planar phenyl rings of curcumin stacks with the planar rings of the aromatic amino acid residues of proteins, thereby altering the conformation and their activity. Human RNase L (741 a.a.) has total of 14 cysteine (7 cysteines forming a cysteine-rich region in the PK-homology domain (293 to 444 a.a.) residues probably making disulphide linkage in the native molecule and 53 aromatic (28 phenylalanine, 7 tryptophan, and 18 tyrosine) a.a. residues, making it a target for curcumin-mediated inhibition. The molecular docking study performed by us clearly indicates that curcumin fits strongly into the hydrophobic pocket (site 4) of human RNase L formed by “**F**FWT**WE**” (aromatic amino acids 585 and 589 that bind curcumin are shown in bold) having two tryptophans and two phenylalanines (Figure 5). The occurrence of closely spaced glutamic acid (E) at position 590 (adjacent to Trp-589) and aspartic acid (D) at positions 575 and 579 might be responsible for such strong fluorescence quenching due to presence of their carboxylate groups. The second predicted site (site 2) for curcumin binding within amino acids Leu-278 to Lys-362, which lies within the region that stabilizes the crossed homodimeric structure of human RNase L [16]. Thus, the higher affinity of curcumin to the hydrophobic site 4, which lies very close to the ribonuclease domain (key active site residues His-672, Arg-677, Asn-678, and Tyr-655) [16]. might perturb or block the active site region at lower curcumin concentrations (5 *μ*M and 10 *μ*M). The fluorescence quenching experiment revealed that there was saturation in curcumin binding and blue shift in fluorescence maxima from 4 *μ*M to 8 *μ*M curcumin, which supported our RNA-degradation assay with maximum activity at 5 *μ*M and as the concentration of curcumin was increased to saturating concentration, namely, 16 *μ*M in intrinsic fluorescence experiment, there was shift in fluorescence maxima to higher wavelength (337 nm from 331 nm at 8 *μ*M) (Figure 4(a)) close to the native (340 nm) and decrease in inhibition by curcumin from 5 *μ*M to 40 *μ*M in RNA-degradation assay (Figure 3(c)). This clearly indicated that the enzyme RNase L has undergone a conformational change such that different residues (or the same residues, but in different environment) have been affected by increasing curcumin binding. Simultaneous binding of curcumin to site 2 might relieve the inhibition first, but as the concentration is increased above 50 *μ*M, there might be major switching in the conformation leading to complete loss in the activity of human RNase L rendering it approximately 77% inactive. Further, higher concentrations of curcumin, that is, 100 and 150 *μ*M, cause inhibition of 85% and 90%, respectively, probably due to disruption of the crossed homodimeric structure at site 2 by curcumin. Curcumin binds to human RNase L with submicromolar affinity and in none of the docking solutions did it bind to the cysteine rich region (7 cysteines present from amino acid 293 to 444) of the kinase homology (KH) domain of human RNase L. The biphasic response might complicate the use of curcumin as a therapeutic agent, but since curcumin can be consumed at higher levels in the diet and only 150 *μ*M curcumin can almost fully inhibit RNase L, it is a very good candidate for development of efficacious curcuminoids in treating RNase L-mediated immune deregulations. However, our data indicates that curcumin is a potent inhibitor of purified human RNase L at submicromolar levels, which might have important physiological consequences particularly for some types of chronic inflammation; it may possibly be used as therapeutic approach to treat RNase L abnormalities, for example, CFS, colorectal carcinogenesis, scrapie infection in brain, and other defects of the RNase L-pathway.

## 5. Conclusion

Curcumin, a natural plant-derived antioxidant, inhibits recombinant GST-RNase L enzymatic activity* in vitro*.

## Figures and Tables

**Table 1:** Results of the molecular docking experiment (number of poses having binding energy).

S. number	Between −4 and −5 kcal/mol	Between −5 and −6 kcal/mol	Between −6 and −7 kcal/mol	Between −7 and −8 kcal/mol	Greater than −8 kcal/mol
Site 1	8	22	114	6	0
Site 2	0	87	49	14	0
Site 3	5	78	67	0	0
Site 4	0	0	29	103	18
Site 5	0	89	61	0	0

Clustering of highest number of poses with binding energy greater than −7 kcal/mol occurs mostly at sites 4 and 2, respectively.
